# Herpes Zoster Ophthalmicus: A Devastating Disease Coming Back with Vengeance or Finding Its Nemesis?

**DOI:** 10.18502/jovr.v17i1.10177

**Published:** 2022-01-21

**Authors:** Michael Tsatsos, Ioannis Athanasiadis, Athina Myrou, George M Saleh, Nikolaos Ziakas

**Affiliations:** ^1^Department of Ophthalmology, Aristotle University of Thessaloniki, Thessaloniki, Greece; ^2^Moorfields Eye Hospital, NHS Foundation Trust at Bedford Eye Clinic, Bedford, UK; ^3^1 st Propeudeutic Internal Medicine Department, AHEPA University Hospital, Thessaloniki, Greece; ^4^National Institute for Health Research Biomedical Research Centre at Moorfields Eye Hospital and the UCL Institute of Ophthalmology, London, UK

**Keywords:** Eye, Herpes Zoster, Immunity, Vaccine

## Abstract

Herpes zoster ophthalmicus is a frequent, painful, and debilitating condition caused by the reactivation of the varicella-zoster virus alongside the ophthalmic branch of the trigeminal nerve. Twenty-five percent of adults will develop the disease during their lifetime with the risk increasing to one in two over the age of 50. Herpes zoster ophthalmicus presents with a plethora of ocular manifestations ranging from the characteristic rash in the distribution of the ophthalmic branch of the fifth cranial nerve to more severe keratouveitis, disciform keratitis, and even retinal necrosis. Up to 20% of affected patients develop post-herpetic neuralgia which can persist for years after the acute episode, resulting in potentially devastating consequences for the patient's social, financial, and professional circumstances, as well as their quality of life and daily activities. Shingles prevention studies indicated that the herpes zoster vaccine markedly reduces the burden of the disease, as well as the incidence of both infection and post-herpetic neuralgia. Here we review the vaccinations available for herpes zoster, the reasons behind their limited adoption so far, as well as the future perspectives and challenges associated with this debilitating disease in the era of herpes zoster vaccination and coronavirus disease pandemic.

##  INTRODUCTION

The varicella zoster virus (VZV) is a highly contagious alpha-herpesvirus that causes two separate diseases in humans: varicella (chickenpox) and herpes zoster (HZ, shingles). Varicella is the primary disease and leads to latency of the virus, primarily in peripheral autonomic ganglia including dorsal root ganglia, cranial nerve ganglia such as the trigeminal ganglion, and autonomic ganglia such as in the enteric nervous system.^[1,2,3]^

**Figure 1:**
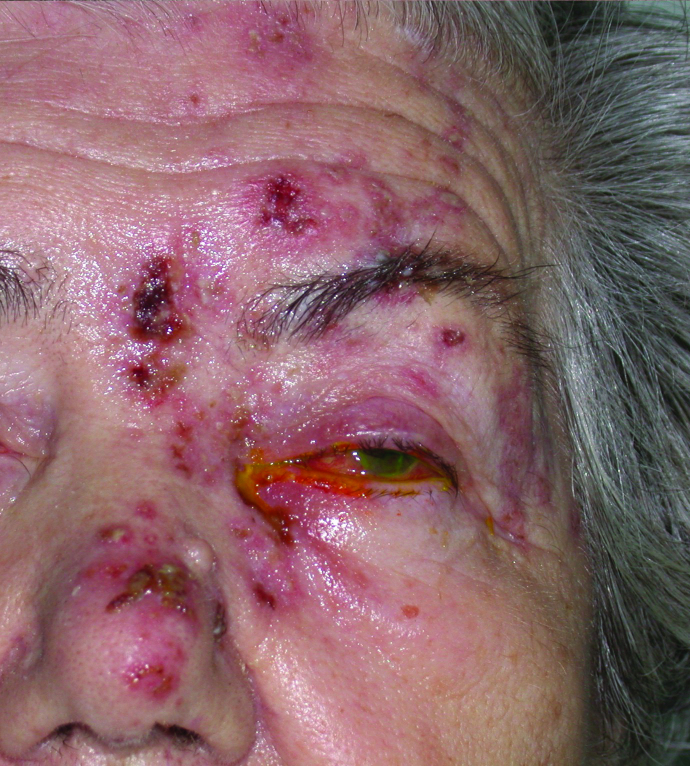
Herpes zoster ophthalmicus with typical dermatomal distribution on the face along the first and second branch of the trigeminal nerve.

Herpes zoster is the secondary disease that results from reactivation of the dormant virus, even decades after the initial infection, either spontaneously or secondary to a number of triggering factors. This usually appears as painful or pruritic cutaneous vesicules that occurs in a certain dermatomal distribution pattern, either on the face or on the back [Figure 1]. This viral reactivation occurs mostly with increasing age due to reduced immunity in this population. Triggering factors that can reactivate the virus also involve immunosuppression from disease or drugs, injury, X-ray irradiation, infection, and malignancy. Approximately one in three people will be affected by HZ during their life.^[1,2,3]^

Varicella zoster virus-related diseases can lead to serious ocular morbidity, which can range from asymptomatic corneal scars to severe sight impairment and in more advanced cases painful blind eyes. An important and potentially devastating complication of shingles, post-herpetic neuralgia, can persist long after the resolution of the rash and can significantly affect patient's quality of life, especially in the population over the age of 60 where other ocular and systemic comorbidities may be present as well. The economic impact of such a debilitating disease cannot be overlooked; loss of working hours, time off work, patient's frequent need of home care and the chronicity of symptoms, and thus treatment, add further financial burden to an already overstretched healthcare system worldwide.^[4]^ Several treatments exist for herpes zoster, but to be successful need to be applied early on the course of the disease. This in turn led to the development of prevention strategies with vaccines.^[1,5]^

### Reasons for Limited Introduction of VZV Vaccinations

Although the health and economic benefits associated with prevention of HZ infections are obvious, the introduction of varicella prophylaxis through vaccination has been a matter of controversy. Effective vaccines against varicella and HZ are available; however, there are healthcare systems that are reluctant to introduce routine vaccination because modelling studies have predicted that the reduction in varicella would lead to an increased incidence of HZ cases.^[2]^ The question as to whether the varicella vaccine results to a higher incidence of shingles remains controversial but has gained popularity through the theory of reduced immune response boosting compared to the actual disease. However, this notion is not widely accepted by the scientific community.^[1]^

Whilst the incidence of zoster is increasing in the United States, this rise began before the varicella vaccine was introduced. Zoster is also increasing in areas where the varicella vaccine is not being used, and this appears to be multifactorial including increased identification of zoster, an aging population, and the ever growing number of immunocompromised patients including those on biological treatments to control a range of diseases.^[1]^

Cost-effectiveness of HZ vaccine has been previously shown to be favorable and comparable to vaccinations for other diseases, however, the adult coverage remains lower than expected.^[6]^

The COVID-19 pandemic has led to circular type of governmental measures in an attempt to restrict its distribution in the community. Thus, a number of HZ patients that would seek help from either primary or secondary care, were reluctant to visit their physician in the midst of the pandemic.^[7]^With the high penetrance of the Delta COVID-19 variant seen in most countries some sort of restriction of movement is here to stay for longer than initially expected, making the case for prevention of any disease a lot more favorable than before.^[8]^

The Shingles Prevention Study, a randomized, placebo-controlled trial, assessed burden of illness and post-herpetic neuralgia (PHN) incidence in > 38,000 people aged ≥ 60 years who received the live attenuated zoster vaccine or a placebo. Compared with placebo, vaccination significantly reduced the severity of HZ cases as well as the incidence of HZ and PHN. The conclusion was that prophylactic vaccination can positively affect the incidence and course of HZ disease and result in an overall improvement of the patient's quality of life.^[5]^

### Available Vaccinations

Nowadays there are two vaccines available for the prevention of HZ, the live attenuated ZostavaxⓇ vaccine (ZVX) first released in 2006 and the newer adjuvanted HZ subunit Shingrix vaccine (HZsu) becoming available almost a decade later, in 2017.^[9]^

In 2008, the Advisory Committee on the Immunization Practices (ACIP) of the United States Centers for Disease Control and Prevention (CDC) recommended the routine vaccination with ZVX for all persons older than 60 years with a dose of the vaccine. Those who report a previous episode of zoster as well as people with chronic medical conditions (e.g., diabetes mellitus) could also be vaccinated. There was no need to consider history of varicella (chickenpox) or to conduct serologic testing for varicella immunity before routine administration of zoster vaccine. ZostavaxⓇ vaccine vaccination at that time was not recommended for people who have received varicella vaccine (VV) in the past.

However, there was no need to ask about VV history before administering ZVX as those eligible, that have received the VV previously, would have been very few. The specific vaccine was introduced toward the end of the previous decade and very few adults were since then eligible for this. Hence, almost all persons in the age group recommended to have ZVX in 2008 would not have received the VV.

Accordingly, the 2018 ACIP recommendation was that HZsu may be used in adults aged ≥ 50 years, irrespective of previously receiving varicella vaccine or ZVX. Also screening for a history of chickenpox (varicella) was not required and adults previously affected by herpes zoster should also receive the vaccine as the disease can recur.^[10,11]^

The live attenuated vaccine (ZVX) ZostavaxⓇ (Merck and Co.) is associated with protection against shingles and PHN in half or more of individuals over 60 years old. Such protection however, wanes over time, starting as early as the first year following immunization and essentially disappearing after eight years. The use of boosters is not recommended in the case of ZostavaxⓇ. The safe use of ZostavaxⓇ is also not guaranteed in immunocompromised persons due to the higher risk of serious VZV infections.^[1]^ Injection site reactions such as pain, swelling, and erythema occur in > 45% of vaccinated people. Headache and more serious adverse events, including hypersensitivity reactions range from uncommon to rare.^[12]^

In an attempt to find an alternative vaccine offering better protection and ensuring the safety of the immunocompromised patients, a new vaccine, Shingrix, was developed (Glaxo Smith Kline). *This *is a non-live “subunit” recombinant vaccine (HZ/su), made of a truncated form of the VZV glycoprotein E surface antigen, combined with the AS01B Adjuvant System, which enhances the immune response to VZV.^[1,3,13]^ The vaccine requires two doses, two to six months apart and provides about 97% protection to healthy persons up to the age of 70 when immunized. It also provides protection against the difficult-to-treat PHN.^[1]^

HZ/su offers 97% protection against HZ in those aged 50 years or older, including 87% efficacy in those 80 years or older, indicating that the efficacy of HZ/su is not greatly affected by the vaccinated individual's age.^[14]^ It is currently being tested for safety and immunogenicity in immunocompromised patients. The most challenging aspect of Shingrix is that is associated with a higher incidence of side effects for the first few days after immunization, such as local skin reactions at the injection site, fever, and malaise. Serious adverse effects are relatively rare.^[1]^

### Disadvantages and Benefits of Available Vaccinations

Advantages and disadvantages of available vaccination are summarized in Table 1. The live attenuated zoster vaccine boosts VZV-specific cell-mediated immunity in older vaccinated individuals, thereby explaining the efficacy of the vaccine. Despite this, efficacy against HZ is limited to 51% in those vaccinated aged 60 years or older, and decreases as the age at the time of vaccination increases. In addition, the protection by ZVX falls significantly 6–8 years after vaccination. The magnitude and duration of protection have been confirmed by effectiveness studies.^[14]^

ZOE-50 and ZOE-70 studies concluded that the recombinant zoster vaccine reduced the incidence of HZ by over 90% and PHN by at least 89% in all studied age groups for at least four years after vaccination. Local and systemic reactions were of mild to moderate intensity and transient in nature. The overall safety profile of the vaccine was clinically acceptable.^[3]^

The public health impact of *Shingrix *vaccination was assessed in a mathematical model, suggesting that in the US, using this vaccine in those ≥ 50 years of age could prevent 11 to 15 million cases of herpes zoster and 1.6 to 2.1 million cases of PHN. Overall, *Shingrix *recombinant vaccine has a clinically acceptable safety profile and a high efficacy against herpes zoster in adults 50 years of age or older.^[3]^

Thirty percent of the unvaccinated adults will develop HZ during their life; increasing to 50% in people ≥ 85 years old. Ophthalmologists worldwide are well aware of the serious HZ-related ophthalmic complications. However, as shown in a large population cohort in Korea, HZ increases the risk of stroke and myocardial infarction, especially in those relatively young who are at less risk for atherosclerosis.^[13]^ Vaccination with *Shingrix *could therefore reduce the incidence and associated costs of herpes zoster and its complications.^[3]^

Another important aspect of Shingrix vaccine that is of outmost significance during the pandemic is that it has been postulated that Shingrix vaccine may help body's immune system against other infections including 2019 coronavirus disease (COVID-19).^[15]^

In view of the health challenges caused by the COVID-19 pandemic and its resultant pressure on healthcare systems worldwide and restriction of movement even for elective health visits, any benefit offered would be welcomed. This synergistic and additive beneficial effect could offer added value if possibly the HZ vaccine is given before the COVID-19 vaccine so as to get the maximum benefit.

**Table 1:** Risks and benefits of zoster vaccination


**Attributes/Vaccine**	**Adantages**	**Disadvantages**	**Risks/Side effects**
**ZOSTAVAX**	No boosters required Protection against Herpes Zoster and Post-Herpetic Neuralgia	Efficacy against Herpes Zoster limited to 51% in vaccinees ≥ 60 years of age Efficacy lower as the age at the time of vaccination increases The protection provided by Zoster Vaccine declines significantly at 6–8 years after vaccination	Injection site reactions Erythema Pain Swelling Pruritus Warmth Hematoma Headache Hypersensitivity reactions (including anaphylactic reactions, fever, rash, and lymphadenopathy at the injection site)
**SHINGRIX**	Protection against Herpes Zoster and Post-Herpetic Neuralgia Reduces incidence of Herpes Zoster by over 90% and post-herpetic neuralgia by at least 89% Efficacy was sustained over at least 4 years after vaccination	Two doses required	Mild to moderate and transient injection site and systemic reactions Fever Malaise Myalgia

**Figure 2:**
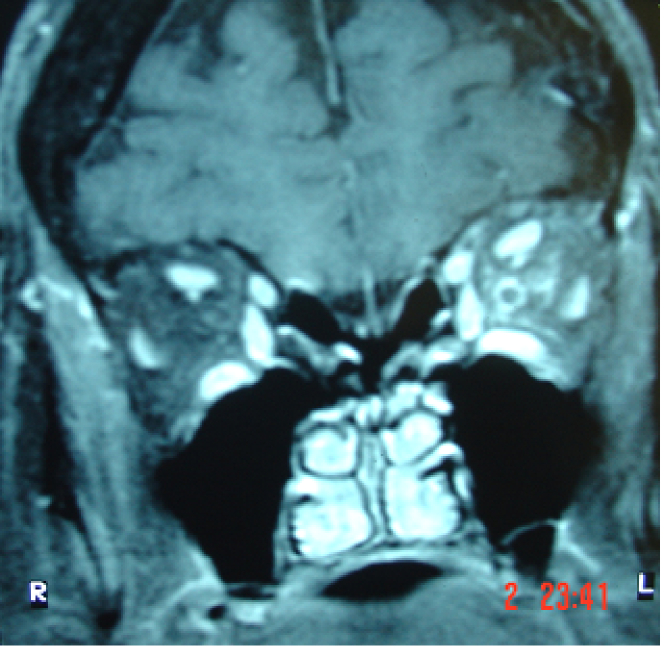
MRI scan depicting characteristic and rare Optic perineuritis resulting from orbital involvement in a patient with Herpes zoster ophthalmicus. Enlargement of extraocular muscles is also obvious on the left side.

### Future Perspectives

Herpes zoster ophthalmicus is associated with serious sequelae locally and systemically that have severe effects on the patients' quality of life and even lifespan. HZO can lead to complications ranging from periocular and conjunctival involvement to the devastating results of multiple ocular and extraocular manifestations [Figure 2]. Visual compromise can occur in severe and recurrent cases; and cases refractory to treatment are not an uncommon encounter to the anterior segment specialist.^[16]^ Whether vaccination can offer a viable solution to help limit the extent of herpes zoster-related complications needs further scientific evaluation so that to better elucidate exactly what the role of vaccination will be; even if this means that herpes zoster ophthalmic disease could become an essentially subspecialist condition requiring referral to tertiary centers. In order to reach the desirable objective, we feel that collaboration with other specialties, such as otorhinolaryngology and neurology, while dealing with the various HZ manifestations is very desirable and could benefit our patients globally.

Recently, following the COVID-19 pandemic, there was special interest shifted on the relation between HZV and Severe Acute Respiratory Syndrome Coronavirus 2 (SARS-CoV-2), and their respective immunization methods. There were reports that vaccination against COVID-19 as well as the disease itself could lead to reactivation of VZV and recurrence of HZV disease in different dermatomes. This could affect both immunocompetent individuals without risk factors or comorbidity that would contribute to the development of HZ disease and immunocompromised patients with autoimmune inflammatory diseases.^[17]^ Similar observations have been previously made in patients suffering from COVID-19, with an increase in HZ cases during the COVID-19 pandemic, which suggests an association between these diseases.^[18]^ Although this correlation is not well-established, this is a field which will definitely attract attention in the future. Indeed, there are already studies underway looking into the association and measuring the effect of the Shingrix vaccine on the immune system and whether that has any effect on the ability to fight off other infections such as COVID-19.^[15]^ With the accumulation of further data, more definite conclusions can be drawn, and strategies can develop to reduce treatment burdens for patients and the healthcare systems overall.

We are now developing the tools which will allow us to take action in preventing the extent and complications of this potentially debilitating, sight threatening or even life threatening disease. The efficacy of Shingrix has been shown to be higher than the previously available vaccine, reaching levels of 97.2% and 91.3% in adults 50 and 70 years, respectively, whereas the live attenuated virus vaccine (Zostavax) has reached around 55% of efficacy.^[13]^

In summary, pediatric/adolescent vaccination against varicella zoster has not been described although an increasing number of infections have been described for both herpes simplex as well as herpes zoster in adolescents and young adults.^[19,20]^ Adult vaccinations prevent morbidity, disability and death and have favorable cost-effectiveness profiles. Efforts to increase the implementation of vaccination in adults and addressing barriers to implementation are needed.^[5,6]^

##  Acknowledgements

George M Saleh was supported by the National Institute for Health Research (NIHR) Biomedical Research Centre based at Moorfields Eye Hospital NHS Foundation Trust and UCL Institute of Ophthalmology. The views expressed are those of the author(s) and not necessarily those of the NHS, the NIHR or the Department of Health.

##  Declaration of patient consent

The authors certify that they have obtained all appropriate patient consent forms. In the form the patient has given her consent for her images and other clinical information to be reported in the journal. The patient understand that her name and initial will not be published and due efforts will be made to conceal her identity, but anonymity cannot be guaranteed.

##  Financial Support and Sponsorship

Nil.

##  Conflicts of Interest

The authors do not have any conflicts of interest. Informed consent allowing the use of face photos for research, publication and teaching has been obtained alongside departmental guidelines.
